# Supplementary material for: Cell-free assays reveal that the HIV-1 capsid protects reverse transcripts from cGAS immune sensing
Source: PLoS Pathog. 2025 Jan 28;21(1):e1012206. doi: 10.1371/journal.ppat.1012206 (PMC11793794; doi:10.1371/journal.ppat.1012206)
Supplement: S1 Table — (PDF) [file ppat.1012206.s001.pdf]

S1 Table. Reagents used in this study.

REAGENT	Source	Catalogue #
Antibodies		
cGAS	Cell Signaling	Cat# 79978<br>RRID:AB_2905508
STING	Cell Signaling	Cat# 13647<br>RRID:AB_2732796
IRF3	Cell Signaling	Cat# 11904<br>RRID:AB_2722521
PQBP1	Proteintech	Cat# 16264-1-AP<br>RRID:AB_10792928
ISG15-PE	R&D Systems	Cat# IC8044P
SIGLEC1-647	BD Biosciences	Cat# 565295<br>RRID:AB_2739163
Actin	BD Biosciences	Cat# 612656<br>RRID:AB_2289199
IRDye 680RD Donkey anti-Mouse fluorescent antibody	LI-COR	Cat# 926-68072<br>RRID:AB_10953628
IRDye 800CW Goat anti-Rabbit fluorescent antibody	LI-COR	Cat# 926-32211<br>RRID:AB_621843
Bacterial and virus strains		
Stbl3 competent *E. coli*	Thermo Fisher	Cat# C737303
BL21-CodonPlus (DE3)-RIPL *E. coli*	Agilent	Cat# 230280
BL21-CodonPlus (DE3)-RIL *E. coli*	Agilent	Cat# 230245
HIV-1 SG3ΔENV	NIH AIDS Reagent Program	Addgene plasmid #11051
HIV-1 SG3ΔENV RT D185A	Christensen et al., 2020	Addgene plasmid #145782
HIV-1 SG3ΔENV CA E45A	Christensen et al., 2020	Addgene plasmid #145783
HIV-1 SG3ΔENV CA Q63/67A	Christensen et al., 2020	Addgene plasmid #145784
HIV-1 SG3ΔENV CA M66I	Christensen et al., 2020	Addgene plasmid #149687
HIV-1 SG3ΔENV CA Q67H/N74D	This study	Addgene plasmid #217442
HIV-1-GFPΔENV	This study	Addgene plasmid #217437
HIV-1-GFP CA E45A	This study	Addgene plasmid #217438
HIV-1-GFP CA Q63/67A	This study	Addgene plasmid #217439
HIV-1-GFP CA M66I	This study	Addgene plasmid #217440
HIV-1-GFP CA Q67H/N74D	This study	Addgene plasmid #217441
pSIV3+	Mangeot et al., 2000	N/A
Chemicals, peptides, and recombinant proteins		
Melittin	Sigma Aldrich	Cat# M2272-5MG
IP6	Sigma Aldrich	Cat# I5125-50G
rATP	Promega	Cat# E6011
rGTP	Promega	Cat# E6031
rCTP	Promega	Cat# E6041
rUTP	Promega	Cat# E6021
dATP	Promega	Cat# U120D
dGTP	Promega	Cat# U121D
dCTP	Promega	Cat# U122D
dTTP	Promega	Cat# U123D

HEPES	Gibco	Cat# 15630080
Phosphate Buffer Saline (PBS)	Gibco	Cat# 14190250
EDTA 0.5M	Invitrogen	Cat# AM9260G
Penicillin-Streptomycin	Thermo Fisher	Cat# 15140-122
Chloramphenicol	Thermo Fisher	Cat# B20841.22
2-Mercaptoethanol	Thermo Fisher	Cat# 21985023
MEM non-essential amino acids	Thermo Fisher	Cat# 11140050
RPMI 1640 medium	Thermo Fisher	Cat# 11875-119
DMEM	Thermo Fisher	Cat# 11995073
Ficoll-Paque Plus	GE Healthcare	Cat# 17-1440-02
Fetal Bovine Serum	Gibco	(Lot #1982147)
Bovine Serum Albumin Fraction V, heat shock	Sigma Aldrich	Cat# 03116956001
Recombinant Human IL-4	Miltenyi Biotech	Cat# 130-093-922
Recombinant Human GM-CSF	Miltenyi Biotech	Cat# 130-093-867
poly-L-lysine hydrobromide	MP Biomedicals	Cat# 0219454405
Polyethylenimine “Max”, (Mw 40,000) - High Potency Linear PEI	Polysciences, Inc.	Cat# 24765-1
Paraformaldehyde	Electron Microscopy Sciences	Cat# 15713-S
Polybrene	Sigma	Cat# TR-1003-G
Puromycin	Invivogen	Cat# ant-pr-1
Halt protease and phosphatase inhibitor	Thermo Fisher	Cat# 78441
Efavirenz	Selleck Chemicals	S4685
Elvitegravir	Selleck Chemicals	S2001
Lenacapavir	MCE	HY-111964
GS-CA1	Gilead	N/A
PF-74 (PF-3450074)	MCE	HY-120072
BOLT transfer buffer (20X)	Invitrogen	BT00061
Novex NuPAGE MOPS SDS Running Buffer (20X)	Invitrogen	NP0001
NuPAGE LDS Sample Buffer (4X)	Invitrogen	NP0007
Benzonase	Sigma	Cat# E1014
Subtilisin Protease from Bacillus	Sigma	Cat# P5380
PMSF	Roche	Cat# 10837091001
NaCl	Thermo Fisher	Cat# BP358-10
Tris-HCl	Thermo Fisher	Cat# 228030051
Tris-base	Thermo Fisher	Cat# BP152-500
$MgCl_2$	Thermo Fisher	Cat# 41341-5000
Isopropyl ß-D-1-thiogalactopyranoside	GoldBio	Cat# I2481C50
2xYT media	Sigma	Cat# Y1003
Imidazole	Thermo Fisher	Cat# AC122020020
Lysozyme	GoldBio	Cat# L-040-100
Pepstatin	Sigma	Cat# 11524488001
Leupeptin	Sigma	Cat# L2884
Aprotinin	Sigma	Cat# 10981532001
DNase I	GoldBio	Cat# D-300-500
His_6-ULP1 protease	Produced in-house	Lao et al. 2018, J. Biol. Chem.
TCEP	Sigma	Cat# C4706
Dithiothreitol (DTT)	Roche	Cat# 10197777001
Ni-NTA resin	Qiagen	Cat# 30210
Casamino acids	Gibco	Cat# 223050
cOmplete His-Tag purification beads	Roche	Cat# 5893682001

EcoRI-HF Restriction Enzyme	New England Biolabs	Cat# R3101S
SYBR Safe DNA Gel Stain	Invitrogen	S33102
TaqMan Fast Universal PCR Master Mix (2X)	Thermo Fisher	Cat# 4352042
Apex General Purpose Agarose	Genesee Scientific	Cat# 20-102GP
Superscript III First-Strand Synthesis System	Thermo Fisher	Cat# 18080051
2X Universal SYBR Green Fast qPCR Mix	Abclonal	Cat# RK21203
Oligonucleotides		
MSSS-FWD (early): AACCCACTGCTTAAGCCTCA	Christensen et al.	N/A
MSSS-REV (early): ACCAGAGTCACACAACAGACG	Christensen et al.	N/A
FST-FWD (intermediate): AGCCGCCTAGCATTTCATCA	Christensen et al.	N/A
FST-REV (intermediate): CCAGCGGAAAGTCCCTTGTA	Christensen et al.	N/A
Late RT-FWD: TGTGTGCCCGTCTGTTGTGT	Christensen et al.	N/A
Late RT-REV: CTTCAGCAAGCCGAGTCCTG	Christensen et al.	N/A
cGAS gRNA target: GAGGCCGCCCTGCCTAAGGC	Johnson et al.	N/A
STING gRNA target: CCCCGTGACCCCTGGGACAC	Johnson et al.	N/A
IRF3 gRNA target: CGTGCGGCTCTTGTTCACCC	Johnson et al.	N/A
PQBP1 KO1 gRNA target: GTCTGCAGCGCAACGGGCAG	This study	N/A
PQBP1 KO2 gRNA target: GGATGCCTCTCTTGGCCAAG	This study	N/A
FST-FWD (HIV-1-GFP, intermediate): AGCCTCCTAGCATTTCGTCAC	This study	N/A
FST-REV (HIV-1-GFP, intermediate): CCAGCGGAAAGTCCCTTGTA	This study	N/A
TaqMan eGFP assay	Thermo Fisher	Cat# Mr04329676_mr
TaqMan IFITM1 assay	Thermo Fisher	Cat# Hs00705137_s1
TaqMan IFITM3 assay	Thermo Fisher	Cat# Hs03057129_s1
TaqMan MX1 assay	Thermo Fisher	Cat# Hs00895608_m1
TaqMan ISG15 assay	Thermo Fisher	Cat# Hs01921425_s1
TaqMan IFI27 assay	Thermo Fisher	Cat# Hs01086373_g1
TaqMan GAPDH assay	Thermo Fisher	Cat# Hs02786624_g1
Recombinant DNA		
psPAX2	Gift from Didier Trono	Addgene plasmid #12260
pCMV-VSV-G	Gift from Bob Weinberg	Addgene plasmid #8454
pCA528	Gift from Wes Sundquist	DNASU plasmid pCA528
pCA528-huPQBP1	This study	Addgene plasmid #217449
lentiCRISPR v2	Gift from Feng Zhang	Addgene plasmid #52961
lentiCRISPR control (LCV2) empty vector minus stuffer	Johnson et al., 2018	Addgene plasmid #217443
lentiCRISPR cGAS	Johnson et al., 2018	Addgene plasmid #217444
lentiCRISPR STING	Johnson et al., 2018	Addgene plasmid #217445
lentiCRISPR IRF3	Johnson et al., 2018	Addgene plasmid #217446
lentiCRISPR PQBP1 KO1	This study	Addgene plasmid #217447
lentiCRISPR PQBP1 KO2	This study	Addgene plasmid #217448

Software		
GraphPad Prism 9.0	GraphPad	https://www.graphpad.com/scientific-software/prism/
FlowJo 10.0	FlowJo LLC	https://www.flowjo.com/
Image Studio Lite 5.5	LI-COR	https://www.licor.com/bio/image-studio-lite/
Morpheus	Broad Institute	https://software.broadinstitute.org/morpheus/
Illustrator	Adobe	https://www.adobe.com/products/illustrator
BioRender	BioRender	https://www.biorender.com/
Other Reagents		
LS Columns	Miltenyi Biotec, Inc	130-042-401
CD14 MicroBeads, human	Miltenyi Biotec, Inc	130-050-201
QuadroMACS Separator	Miltenyi Biotec, Inc	130-090-976
ProFlex PCR System	Thermo Fisher	Cat# A41182
QuantStudio 3 Real-Time PCR System	Applied Biosystems	A28137
Attune NxT Flow Cytometer	Thermo Fisher	A24858
Attune NxT Autosampler	Thermo Fisher	4473928
Odyssey CLx Imager	LI-COR	CLX-2063
SpectraMax ID.5 Plate Reader	Molecular Devices	Cat #76175-288
BioTek Synergy Neo2 Plate Reader	Agilent	BTNEO2
SW 32 Ti Rotor, Swinging Bucket	Beckman	Cat# 369694
0.45μm syringe filters	Corning	Cat# 28200-026
Optima Ultracentrifuge	Beckman	Model: LE-80k
Bolt 4-12% Bis-Tris Plus Gels	Invitrogen	NW04125BOX
HiTrap Heparin HP 5 mL	Cytiva	Cat# 17040701
HiTrap Q HP 5 mL	Cytiva	Cat# 17115401
Superdex 75, 120 mL;16/600	Cytiva	Cat# 28989333
Thinwall Polyallomer, Konical Tubes; 25 x 89 mm	Beckman	Cat# 358126
Open-Top Thinwall Ultra-Clear Tube, 25 x 89 mm	Beckman	Cat# 344058
2',3'-cGAMP ELISA Kit	Arbor Assays	Cat# K067-H5
LIVE/DEAD Fixable Violet Dead Cell Stain Kit	Molecular Probes	L34955
BCA protein assay, reducing agent compatible	Thermo Fisher	Cat# PI23252
p24 ELISA	Xpress Bio	XB-1000
PureLink HiPure Plasmid Maxiprep Kit	Invitrogen	K210007
QIAquick PCR Purification Kit	Qiagen	Cat# 28106
Cytofix/Cytoperm Fixation/Permeabilization Kit	BD Biosciences	554714
Human leukocytes from normal donors (deidentified)	ARUP Components	N/A
DNeasy Blood & Tissue Kit	Qiagen	Cat# 69504
QIAquick Gel Extraction Kit	Qiagen	Cat# 28706
RNeasy 96 Kit	Qiagen	Cat# 74181
Owl EasyCast B1 Mini Gel Electrophoresis System	Thermo Fisher	Cat# B1-BP
PowerEase Touch 250W Power Supply	Thermo Fisher	Cat# PSC350M
iBright FL1500 Imaging System	Thermo Fisher	Cat# A44115
