# Supplementary figures and images for: Cell-free assays reveal that the HIV-1 capsid protects reverse transcripts from cGAS immune sensing
Source: PLoS Pathog. 2025 Jan 28;21(1):e1012206. doi: 10.1371/journal.ppat.1012206 (PMC11793794; doi:10.1371/journal.ppat.1012206)

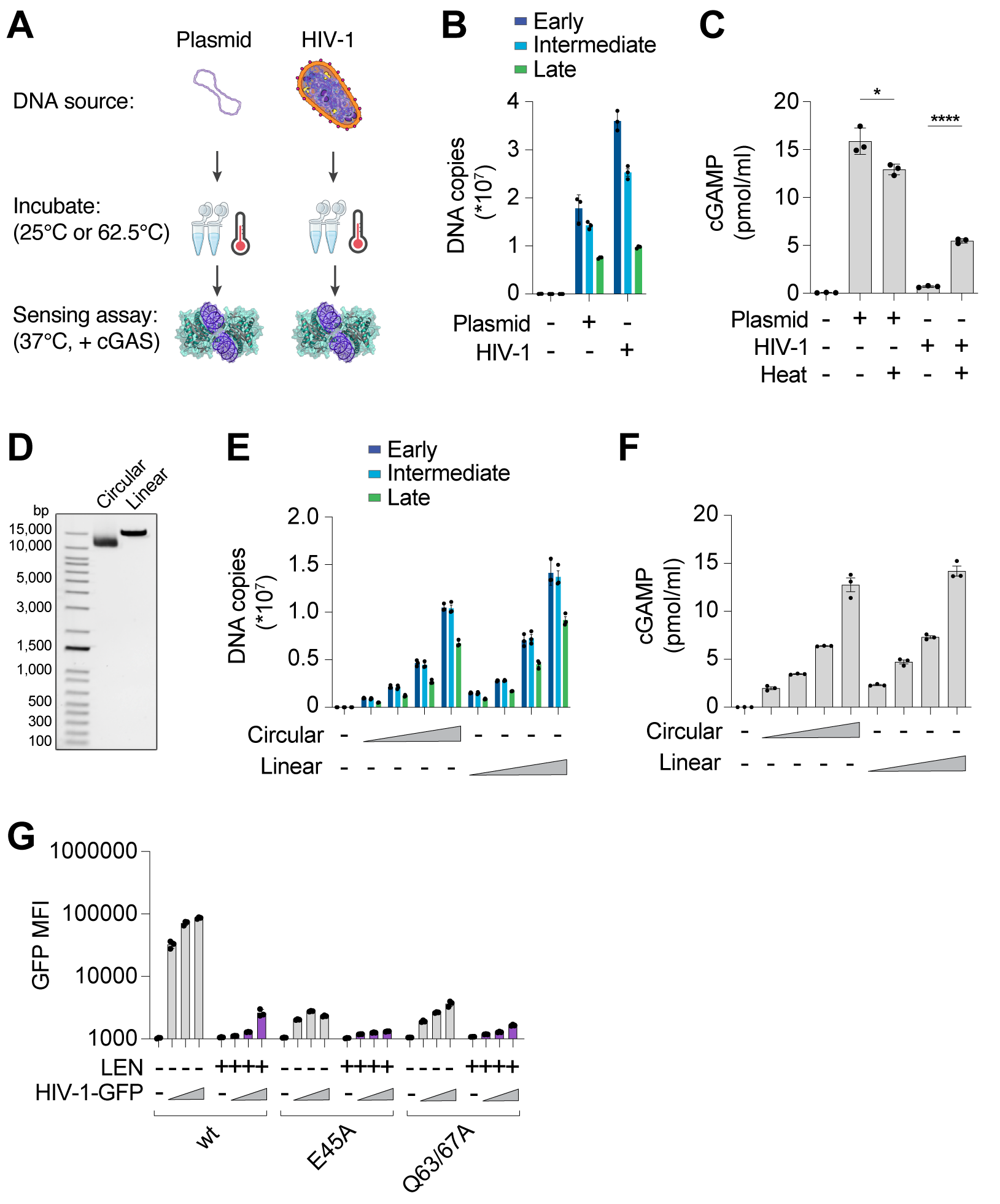

Supplement: S1 Fig — (A) Schematic depicting heat treatment of plasmid or ERT samples prior to cell-free cGAS sensing assays. (B) Copy number of plasmid DNA or viral DNA from ERT reactions that were incubated under identical “standard” conditions (16 h at 37°C) to be taken through limited heat treatment. (C) Cell-free sensing assay (related to panels A&B) comparing the ability of plasmid DNA and HIV-1 ERT samples to activate recombinant cGAS. After ERT, samples were incubated briefly (20 s) at 62.5°C before assaying with cGAS under standard conditions (8 h for 37°C). Heat treatment slightly decreased the ability of plasmid DNA to activate cGAS (possibly through partial denaturation and/or aggregation of the DNA into a less accessible structure). In contrast, heat treatment of ERT products robustly increased cGAS activity. (D) Gel electrophoresis image depicting a molecular weight ladder (lane 1), circular control DNA (pSG3Δenv plasmid, lane 2), and linear control DNA (pSG3Δenv plasmid digested with EcoRI, lane 3). (E) qPCR measurements of DNA copy numbers from circular and linear control DNA samples from (D) that were incubated under standard ERT conditions (16 h at 37°C) in a 2-fold dilution series. (F) Cell-free sensing assay (related to panels D&E) of circular and linear control DNA. Samples were incubated with recombinant cGAS and cGAMP levels were measured by ELISA. (G) GFP mean fluorescence intensity measured by flow cytometry in THP-1 monocytic cells that were infected for 48 h with HIV-1-GFP, the capsid stabilized CA mutant E45A, or the destabilized mutant Q63/67A at matched virus doses (12.5, 25, and 50 nM p24). Where indicated, LEN (10 nM) was added at the time of infection. For (C), statistics were calculated using an unpaired t test to compare plasmid or HIV-1 samples: p < 0.05: *, p < 0.0001: ****. Graphs show mean ± SD from three samples from a representative experiment, selected from two independent experiments. Graphics were generated in Illustrator or created in BioRend [file ppat.1012206.s002.tif]

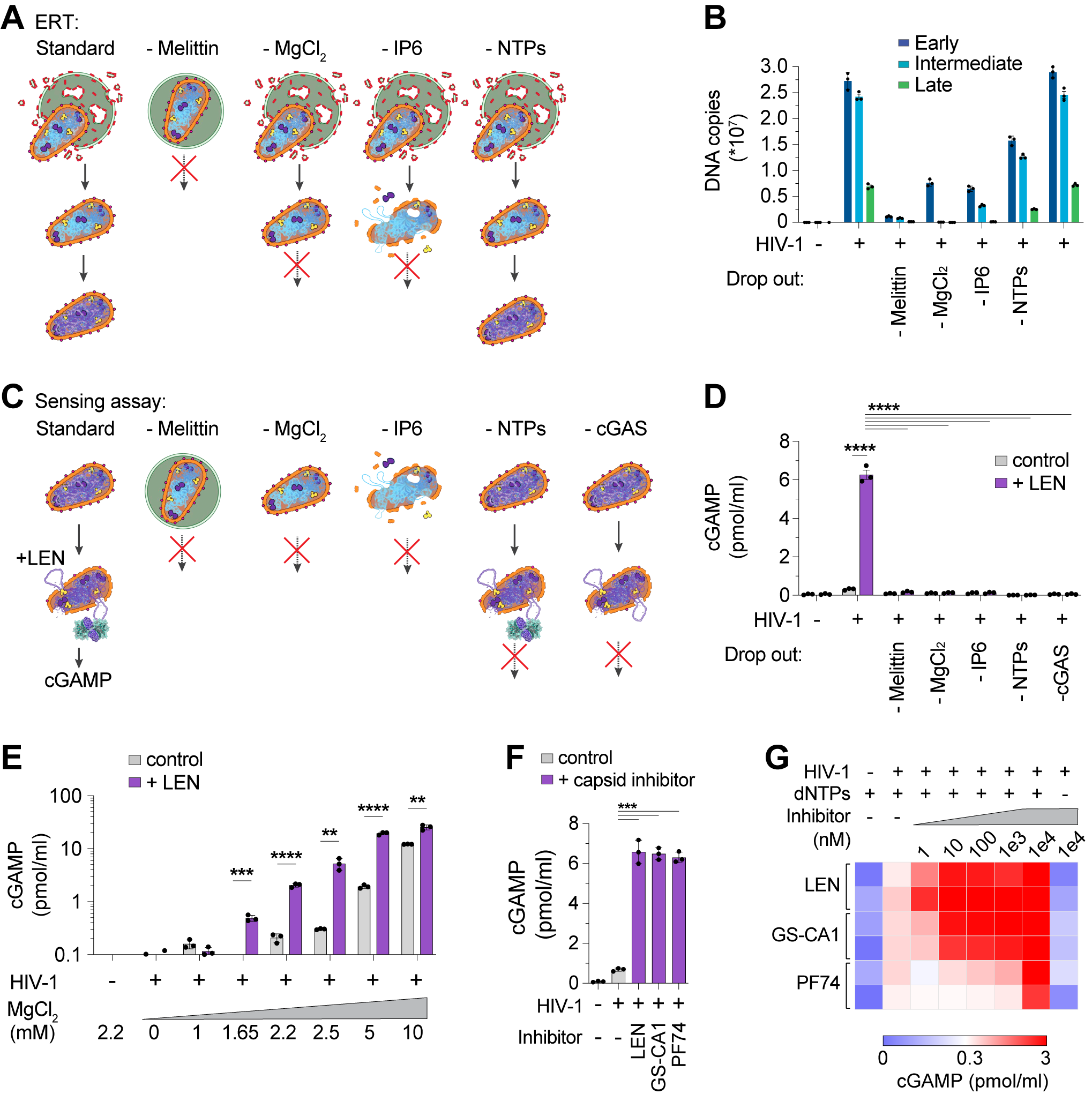

Supplement: S2 Fig — (A) Schematic illustrating that under standard ERT reaction conditions, viral RNA is converted into DNA inside an intact or largely intact capsid. When key components are omitted from the reaction, the ERT reaction is inhibited at the indicated steps. (B) qPCR measurements of DNA copy numbers from ERT reactions that were carried out for 16 h at 37°C under standard conditions (including melittin, MgCl2, IP6, NTPs, dNTPs, and additional buffer components as stated in the Methods) compared to reactions performed in the absence of melittin, MgCl2, IP6, or NTPs. (C) Models for cell-free sensing reactions corresponding to ERT conditions depicted in (A). Core disruption with LEN permits cGAS sensing of RT products under standard reactions conditions. Sensing is blocked at the indicated steps when specific reaction components are removed. (D) Cell-free cGAS sensing assay performed with ERT samples shown in (B). After ERT, samples were incubated with (or without) recombinant cGAS under the conditions shown, with or without LEN (100 nM). cGAMP levels were determined by ELISA. (E) Cell-free sensing assay showing sensitivity to MgCl2 concentration. Standard HIV-1 ERT samples were aliquoted into cGAS reactions with the indicated amounts of MgCl2, and with or without LEN (100 nM). (F) Cell-free sensing assay depicting core disruption by other capsid inhibitors. ERT samples that were prepared under standard conditions (16 h at 37°C) were aliquoted into cGAS reactions and incubated in the presence or absence of LEN (100 nM), GS-CA1 (100 nM), or PF74 (10 µM). (G) Heat map showing dose-dependent increase in cGAS activity for samples incubated with LEN, GS-CA1, or PF74. Samples were prepared for cGAS assays as in (F) with increasing amounts of the indicated capsid inhibitors. Two replicates shown from one of three independent experiments. Statistics were calculated using a one-way ANOVA with Tukey’s multiple comparisons test in (D,F) or an unpaired t test for each concentration of MgC [file ppat.1012206.s003.tif]

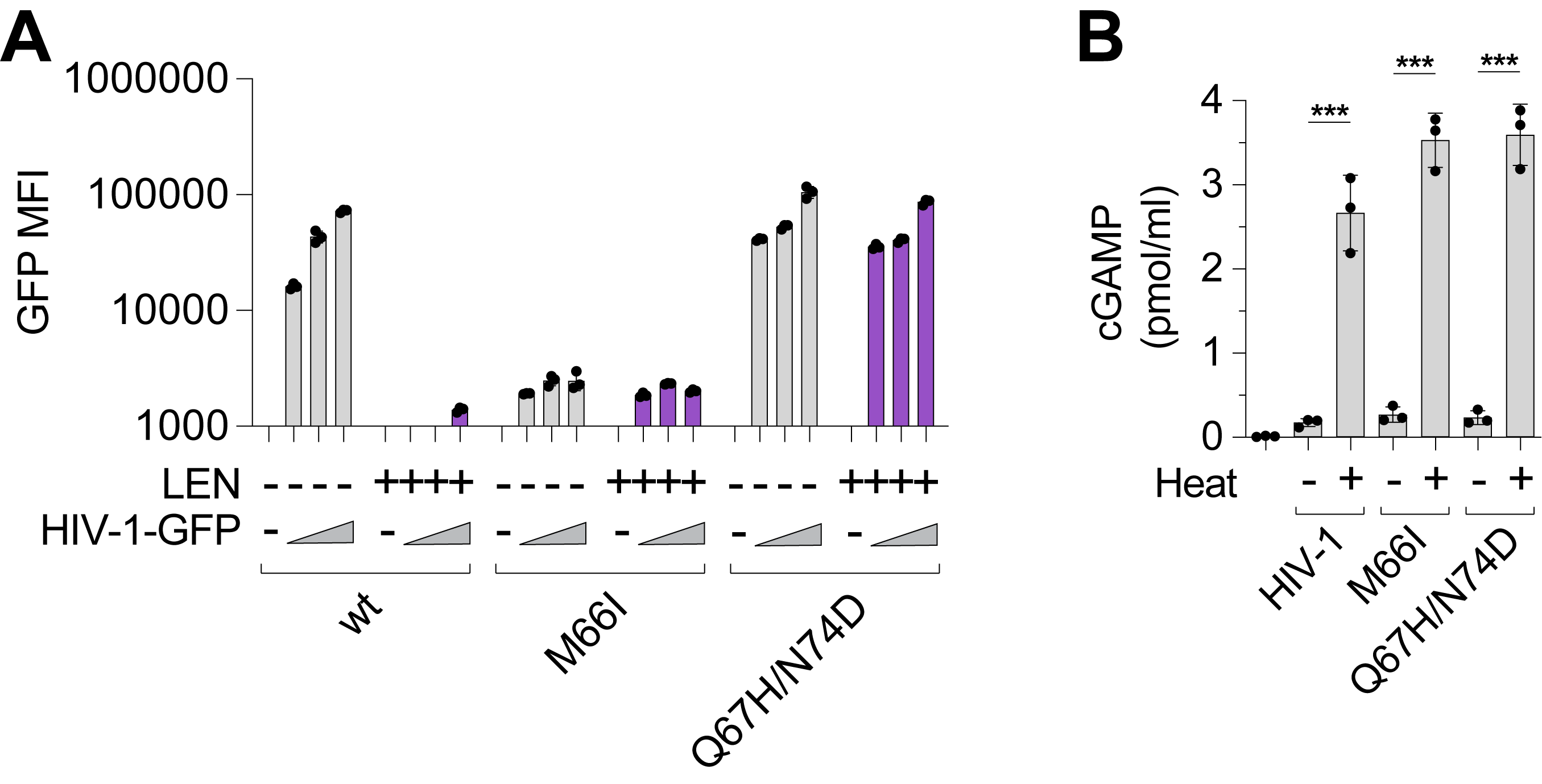

Supplement: S3 Fig — (A) Mean fluorescence intensity of GFP measured by flow cytometry in THP-1 monocytic cells infected for 48 h with HIV-1-GFP compared to LEN-resistant CA mutants M66I and Q67H/N74D at matched virus doses (12.5, 25, and 50 nM p24). Where indicated, LEN (10 nM) was added at the time of infection. (B) Cell-free sensing assay of wt, M66I, Q67H/N74D virions. Samples from ERT reactions shown in Fig 3A that were incubated under standard conditions (16 h of ERT at 37°C) were then briefly heat treated (20 s at 62.5°C) before incubating with recombinant cGAS for 8 h at 37°C. cGAMP levels were measured by ELISA. Statistics were calculated using a one-way ANOVA with Tukey’s multiple comparisons test: p < 0.001: ***. Graphs depict mean ± SD from three samples from a representative experiment, selected from 2 (A) or 3 (B) independent experiments. (TIF) [file ppat.1012206.s004.tif]

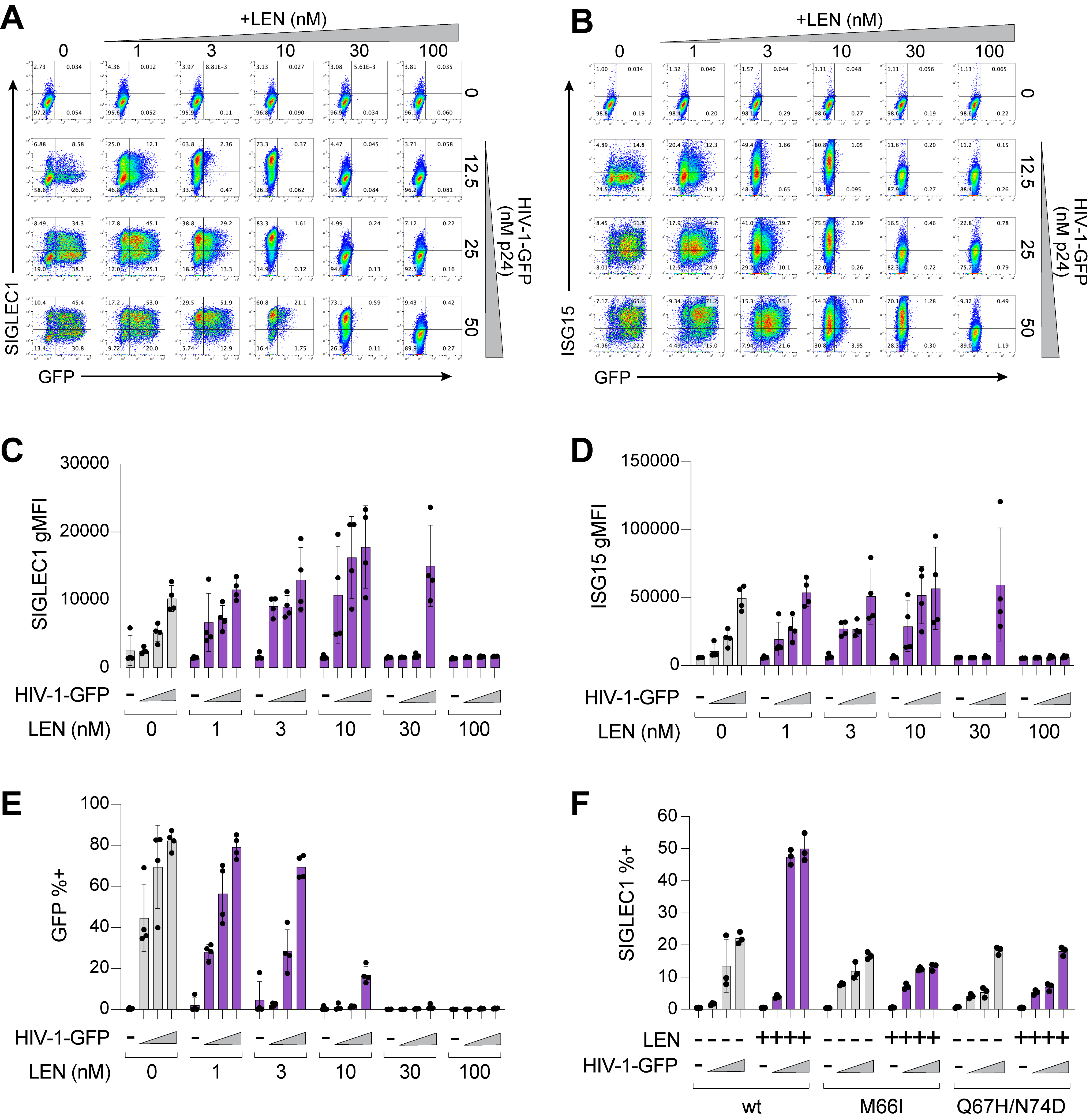

Supplement: S4 Fig — (A and B) Flow cytometry of THP-1 monocytic cells infected for 48 h with a range of HIV-1-GFP doses (12.5, 25, and 50 nM p24) and increasing amounts of LEN. GFP is displayed on the x-axis and either SIGLEC1 (A) or ISG15 (B) are shown on the y-axis. Plots represent one of four infection replicates, which are averaged in the heat maps shown in Fig 4C. (C–E) Flow cytometry data from THP-1 cells infected with a range of HIV-1-GFP doses (12.5, 25, and 50 nM p24) for 48 h and increasing amounts of LEN (0, 1, 3, 10, 30, and 100 nM), showing SIGLEC1 gMFI (C), ISG15 gMFI (D), or %GFP+ (E). (F) Percent SIGLEC1-positive cells measured by flow cytometry. THP-1 monocytic cells were infected for 48 h with HIV-1-GFP compared to LEN-resistant CA mutants M66I and Q67H/N74D at matched doses (12.5, 25, and 50 nM p24). LEN (10 nM) was added at the time of infection. Graphs show mean ± SD from three-four samples from a representative experiment, selected from three (C-E) or two (F) independent experiments. (TIF) [file ppat.1012206.s005.tif]

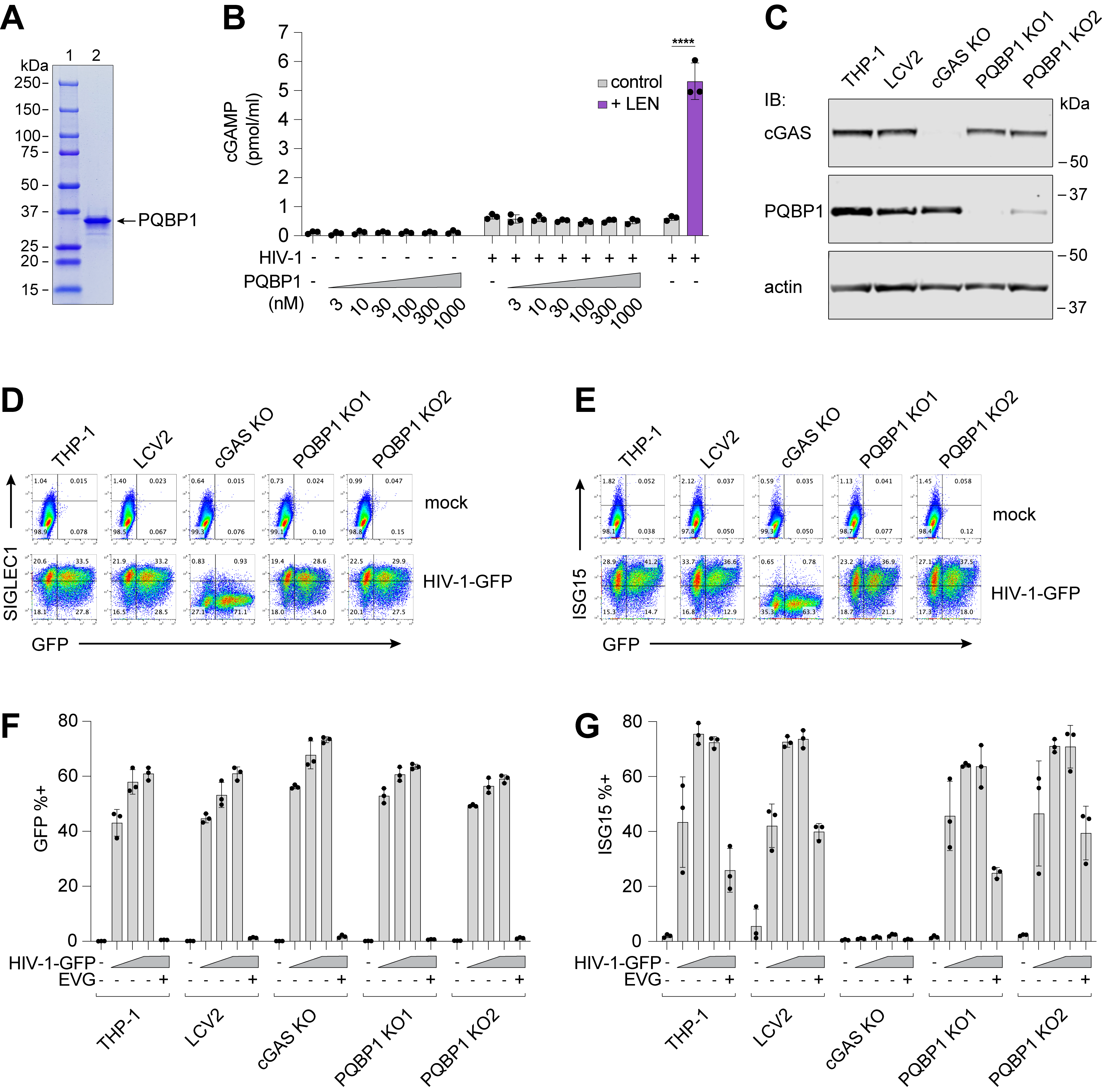

Supplement: S5 Fig — (A) SDS-PAGE with Coomassie staining showing a molecular weight ladder (lane 1) and purified recombinant human PQBP1 protein (lane 2). (B) Cell-free sensing assay to determine cGAS activity in the presence or absence of PQBP1. Purified PQBP1 was incubated at increasing concentrations with recombinant cGAS alone, or together with HIV-1 samples aliquoted from standard ERT reactions (16 h at 37°C). Cell-free sensing assay reactions were performed under standard conditions. LEN (100 nM) was included as a positive control for viral capsid disruption. (C) Immunoblots of THP-1 lysates after lentiCRISPR editing, depicting KO of cGAS or PQBP1 (two independent gRNAs targeting PQBP1), relative to a non-targeting vector control (LCV2). (D) Flow cytometry of lentiCRISPR-modified THP-1 cells infected with HIV-1-GFP for 48 h (50 nM p24), depicting GFP expression on the x-axis and SIGLEC1 on the y-axis. (E) Flow cytometry of lentiCRISPR-modified THP-1 cells infected with HIV-1-GFP for 48 h (50 nM p24), depicting GFP expression on the x-axis and ISG15 on the y-axis. (F and G) Flow cytometry data from THP-1 cells infected with a range of HIV-1-GFP doses (12.5, 25, and 50 nM p24) for 48 h, showing %GFP+ (F) or %ISG15+ (G). To evaluate whether cGAS or PQBP1 have roles in sensing early steps in the virus life cycle, we also tested HIV-1-GFP (50 nM p24) in the presence of the integration inhibitor elvitegravir (EVG, 1 μM). All graphs show mean ± SD from three samples from a representative experiment, selected from two independent experiments. (TIF) [file ppat.1012206.s006.tif]
