# Supplementary material for: Cell-free assays reveal that the HIV-1 capsid protects reverse transcripts from cGAS immune sensing
Source: PLoS Pathog. 2025 Jan 28;21(1):e1012206. doi: 10.1371/journal.ppat.1012206 (PMC11793794; doi:10.1371/journal.ppat.1012206)
Supplement: Attachment — Submitted filename: Response to reviewers PLOS Path 20241110.pdf [file ppat.1012206.s008.pdf]

Response to Reviewers

We thank the editor and reviewers for their thoughtful comments and suggestions for our manuscript. Below, we provide a point-by-point response to reviews, with reviewers' comments in blue and our responses in black.

Dr. Johnson,
Thank you for your patience, reviewers are often a bit late in returning their reviews assigned right around the Cold Spring Harbor Meeting. As you can see, all three reviewers were enthusiastic about the cell free system utilized in your study, which was viewed as innovative and elegant, and I agree. However, the reviewers also noted a number of issues that should be addressed prior to acceptance. Many but not all of these issues are associated with the cellular experiments in your study, with 2 reviewers noting to various degrees the apparent discord between some of your results and previously published studies. While I do not feel that you are obliged to agree with previously published studies in your own study, the reviewers suggest some ways that the timing and dosing of drug or experimental readouts might be modified to allow those in the field to compare other results to the results of your study, and I am inclined to agree some additional experiments in this regard would help accomplish this. Although not noted by reviewers specifically, I also noted that you are achieving a very high level of infection in your THP-1 studies (>80% infection) and would ask that you provide at least one experiment where the cellular responses such as ISG15 or Siglec1 expression are examined at a lower MOI, as I tend to think this is where divergence between study results can occur. In general, I think it is fine for you to use an amount of virus that allows you to monitor the differences you are interested in, so you need not perform every experiment at a lower MOI, but one key result would be useful to those doing research in this area.

We have taken great care to address the issues raised by the reviewers. Most importantly, we have paid careful attention to the MOIs used in our studies and have performed cellular experiments across a range of virus and drug concentrations. We find that therapeutic levels of lenacapavir also potentiate innate immune responses when virus is used at low MOI (Fig 4, new Fig S4, new Fig 5). Importantly, all of these experiments support our original claims and show that lenacapavir can activate innate immune responses under low drug and MOI conditions.

Reviewer's Responses to Questions

Part I - Summary
Please use this section to discuss strengths/weaknesses of study, novelty/significance, general execution and scholarship.

Reviewer #1: The study by Scott et al., utilizes a cell free system, that monitors changes in HIV-1 CA stability and how this affects innate immune sensing of HIV-1 RT products through the cGAS-STING pathway. Shielding the viral genome by the capsid shell to evade host sensing is well documented and so the findings in this study are not novel. However, the cell free system is a great tool to understand changes in CA architecture contributing to innate sensing and productive infection. The authors have done a thorough job in explaining most of their findings with illustrations. The novel part of this study is understanding the mode of action of LEN in abolishing infection and eliciting an immune response following treatment. Expect a few additional experiments and clarifications, this is a well-executed work. As mentioned earlier, the concept of HIV_1 capsid as a shield or molecular cloak protecting the

genome inside is not that novel and therefore incorporating more data that would address other events in the early life cycle such as RT completion, trigger for capsid disassembly would improve the impact of this work.

We thank this reviewer for considering our manuscript to be "a well-executed work" and appreciate our novel finding mode of action for LEN "in abolishing infection and eliciting an immune response." We are actively working to understand other events in the early life cycle and expect that future studies will shed light on the trigger for capsid disassembly. As described below, we have performed additional experiments to test reverse transcription in infected THP-1 cells with a range of lenacapavir concentrations (new Fig 5) and have observed effects that parallel that of our in vitro assays (Fig 4).

Reviewer #2: In this study, Scott et al. report the use of the previously described cell-free reconstituted endogenous HIV-1 reverse transcription system for measuring cGAS sensing of HIV-1 DNA with and without the capsid inhibitor lenacapavir. Interestingly, they show that lenacapavir binding to capsid can promote cGAS activation via the STING pathway in vitro that is similar to results in myeloid cells. This effect was dependent on viral DNA production and was not affected by the presence or absence of PQBP1.

This is a clever system that allows the inclusion of defined factors to interrogate capsid dissociation, reverse transcription, and cGAS sensing. However, the inclusion of several additional measurements, descriptions, and discussion points would improve the manuscript.

We thank this reviewer for their positive comments. We have responded to these suggestions by including additional measurements, descriptions, and discussion points in our revised manuscript (details below).

Reviewer #3: Scott et al. describe an innovative system to measure the sensing of the HIV-1 genome using purified cores. The system is elegantly developed and highly convincing. Additionally, the investigators demonstrated that LEN treatment on purified viral cores triggers the exposure of the viral DNA, which is then sensed by purified cGAS. However, the data developed in cells is problematic and requires further testing. Investigators should correlate core destabilization during infection of cells by LEN with induction of ISGs.

We appreciate that this reviewer considers our cell-free system to be "elegantly developed and highly convincing." In our revised manuscript, we have now correlated the effects of LEN in vitro with its effects in cells and observe progressive phenotypes at increasing concentrations of drug (see Fig 4, new Fig S4, and new Fig 5). At lower concentrations of LEN, we find a potent block to infection that correlates with induction of ISGs, and this occurs prior to any significant adverse impact on reverse transcription. At higher concentrations of LEN, the core is presumably disrupted to the point where reverse transcription is impaired. Remarkably, the concentrations of LEN that inhibit reverse transcription in vitro are identical to those that inhibit reverse transcription in cells (compare Fig 4A with Fig 5A). Perhaps the most significant finding that we report in our revised manuscript is that therapeutic concentrations of LEN (10 nM) dramatically increase innate immune sensing of virus when infections in THP-1 are performed at low MOI (< 0.5), as measured by increased expression of several ISGs by qPCR (*IFITM1, IFITM3, MX1, ISG15*, and *IFI27*; see new Fig 5B).

Part II – Major Issues: Key Experiments Required for Acceptance

Please use this section to detail the key new experiments or modifications of existing experiments that should be absolutely required to validate study conclusions.

Generally, there should be no more than 3 such required experiments or major modifications for a "Major Revision" recommendation. If more than 3 experiments are necessary to validate the study conclusions, then you are encouraged to recommend "Reject".

Reviewer #1: In Fig 1 and Fig S1, the authors bring out the point that CA stability is important to avoid cGAS mediated sensing of viral RT products. In Fig 1G, IP6 at cellular concentrations is sufficient to prevent cGAMP activation in unstable capsids. How does this correspond to viral infection in cells. Is infection restored in unstable capsid at this concentration of IP6?

As Reviewer #1 correctly points out, bulk cellular concentrations of IP6 (~40 μM) are largely sufficient to prevent core destabilization and cGAS activation in vitro for the Q63/67A destabilized capsid mutant (Fig 1G). When we test reporter viruses carrying the Q63/67A mutation in cells, we find that infection is compromised (Fig S1G), as previously reported (Forshey *et al., J Virol*, PMID: 11991995). Since we have not manipulated the concentration of IP6 in cells, we can reasonably surmise that other factors, in addition to IP6, likely interact with this mutant and impede its path in the cell, which agrees with several publications for this and other capsid mutants (Lee *et al., Cell Host & Microbe*, PMID: 20227665; Ohainle *et al., PLOS Pathog*, PMID: 32282853). Thus, infection by unstable capsids is not restored in cells (which, by definition, have cellular IP6 concentrations).

In lines 90-95, the authors state that CA stability can modulate cGAS sensing, but do not comment on whether host factor recruitment is important to maintain the stability such as IP6, CypA etc.

Following this suggestion, we have now commented in the discussion that it is likely that recruitment of host factors in the cell serves to further modify capsid stability.

What is the role of CypA in maintaining CA stability. Can the authors supplement CypA to their assay and see if this avoids cGAS sensing. The results might not be novel but would emphasis on how host factor recruitment is crucial to avoid innate sensing.

This is an excellent idea! We share your interest in this area and are currently working to determine whether CypA functions to maintain HIV-1 core stability. However, we feel that experiments with CypA and other capsid binding factors fall outside the scope of this initial study (as the central messages are that the HIV-1 capsid has the intrinsic property of protecting reverse transcripts from cGAS detection, and that this cloaking can be relieved by lenacapavir treatment). Rigorously testing CypA and other recombinant proteins requires a significant investment of time and resources, and while we hope to work more in this area soon, our efforts for this current manuscript revision have been focused on strengthening our main findings and responding as best we can to reviewers' concerns.

Although the authors talk about viral uncoating and viral dsDNA accumulation as a trigger for this

process, they do not explain in detail the conclusions from Fig S2E. Increasing the concentration of MgCl2 also elevates cGAMP levels in the absence of LEN. Explanation? Does it increase dsDNA accumulation and possibly rupture? It is still unclear how viral capsid disassembles inside the nucleus and the authors possibly have an experiment in there that could shed some insights into this.

Yes, we report in Fig S2E that increasing $MgCl_2$ concentration elevates cGAS activity in the absence of LEN, and this could be a potential mechanism that underpins the release of viral DNA. In our original submission, we provided two possible explanations for this observation in the Methods section: "We speculate that these effects could be driven by two factors: 1) cations such as Mg^{2+} and Mn^{2+} support cGAS activity in vitro[69], and 2) at high concentrations, Mg^{2+} cations might disrupt capsid stability, by interfering with CA subunit interactions and/or by chelating IP6 away from the capsid, leading to loss of capsid integrity, genome release, and reduced responsiveness to LEN." We have now rephrased this explanation and moved it to the Results section to bring more attention to these points. Future work may indeed shed additional insight into how Mg^{2+} or other cations may promote viral core disruption.

Reviewer #2: 1. What is the definition of "early," "intermediate," and "late" reverse transcripts? The proportion of detected DNA called "late" cDNA (i.e. late relative to early/intermediate) is higher for plasmid compared to HIV-1 in Figure 1B. Why is this? And could this account for lower activation of cGAS and production of cGAMP for virus in Figure 1C and S1C? In Figure 1, it appears that the naked DNA control is purified plasmid, which is presumably mostly circular and supercoiled. Would the kinetics and copy numbers of cGAS activation differ if the plasmid was linearized, which is more similar to the dsDNA viral genome?

Primer sets that recognize "early," "intermediate," and "late" reverse transcripts correspond to "minus-strand strong stop," "first strand transfer," and "second strand transfer" events in reverse transcription. These terms are defined the first time they are used in the legend for Fig 1. To be clearer in our revised manuscript, we have now also defined these primer sets in the Methods section. The sequences detected by these primer sets are found in both the plasmid and virus genome, so they can be used to fairly compare DNA copy number between these samples. Plasmid DNA copy numbers for "early" and "intermediate" sequences are roughly double the copy number of "late" sequences because there are two early and intermediate primer binding sites present for these amplicons in the target. As Reviewer #2 correctly points out, the proportion of "late" transcripts is higher for the plasmid than for most virus ERT reactions because: 1) the efficiency of ERT is not perfect, 2) there are two early and intermediate primer binding sites on each copied genome, and 3) RT can initiate on both packaged viral RNA strands (before ultimately resolving to a single copy of late dsDNA). Hence, the signal for "late" reverse transcripts is expected to be less than half the value of "early" or "intermediate" transcripts (just as is observed). Nevertheless, the lower proportion of late RT products in the virus samples does not account for the lower activation of cGAS sensing and cGAMP production for the virus samples in Figs 1C and S1C because: 1) the overall DNA copy numbers for all of the different products (i.e., early, intermediate, and late transcripts) are higher for the virus samples than for the plasmid control, 2) every viral transcript is longer than the minimum size required for cGAS activation, and 3) pretreating cores with heat results in strong cGAS activation (demonstrating that viral dsDNA is present at levels that could be sensed if it were not protected by the capsid, see Fig. 1D).

We also thank this reviewer for pointing out that the purified plasmid we have used is circular/supercoiled and for suggesting this good control experiment. In our revised manuscript we have included tests that compare the circular plasmid to the same plasmid that has been linearized by restriction digest (new Fig S1D). We report that cGAS activity is similar when detecting equivalent copy

numbers of circular versus linear substrate (new Fig S1E-F). This agrees with data from other recombinant cGAS assays in the literature (Mackenzie *et al., Nature*. 2017. PMID: 28738408) reporting similar levels of cGAS activity in response to linear and circular DNA substrates.

2. Some have hypothesized that production of dsDNA during reverse transcription induces capsid uncoating. In Figure 3B, is full-length viral DNA is produced in the majority of the capsids during ERT? If not, could that be the result of a lack of capsid uncoating in this system?

We think this reviewer may be referring to Figures 3C and 3D, where we show ERT for extended amounts of time, out to 72 h, and subsequently test for cGAS activity in the same samples. We find that by 16 h, ERT efficiencies are relatively high, with roughly one out of every two cores generating "late" reverse transcripts. The ERT efficiencies we report here agree nicely with data reported in our previous publication (Christensen et al., Science. 2020. PMID: 33033190). We know from that study that full reverse transcripts are generated by 10 h of ERT because we were able to observe concerted integration of both viral DNA ends into a target plasmid at that time. We are confident that a significant proportion of capsids contain full-length or near full-length genomes by 16 h after initiating ERT and we see no further increase in ERT yields when reactions proceed for 72 h. Since the kinetics of infection in cells are well-established (e.g. uncoating, integration, and expression of new viral transcripts can occur as early as 10-16 h after infection (Burdick et al., PNAS. 2020. PMID: 32094182)), it's unlikely that reverse transcription, on its own, necessitates uncoating. We agree that reverse transcription may generate internal pressure within the capsid and prime uncoating, but our experiments tell us that reverse transcription is insufficient to expose the majority of viral DNA to cGAS.

3. Infectivity data in Figure 4 suggests the STING-mediated IFN-I stimulation can occur during infection in the absence of lenacapavir. Is this a result of full completion of reverse transcription? Or a result of other cellular factors? Movie S1 seems to indicate that capsid uncoating does not occur due to completion of reverse transcription (on a microtubule in the cytoplasm?), but this has not been conclusively demonstrated.

This is an excellent question, but we cannot be sure whether the IFN activation we observe in our original Fig 4 (new Fig 6) is the result of full completion of reverse transcription. At low MOI, only a subset of infected myeloid cells can respond and produce IFN during HIV-1 infection, and so we speculate that cellular factors are involved in triggering cGAS-STING activation in those cells. This concept is further supported by the observation that in T cells, HIV-1 does not trigger an appreciable IFN response, despite having an intact cGAS-STING pathway (Elsner et al., PNAS. 2020. PMID: 32709741), so it's likely that cellular factors have a role in determining stimulation of the IFN pathway.

Regarding Movie S1, yes, it depicts a core undergoing reverse transcription as it traffics on a microtubule but does not specify the location where reverse transcription completes during a successful infection. Movie S1 reflects the central finding from this study, which is that the viral DNA remains largely protected from cGAS, unless the integrity of the core is disrupted by lenacapavir or other means. We understand that this molecular animation may not faithfully represent what happens during an infection or depict exactly how the capsid ruptures in the presence of lenacapavir, but it allows us to visualize one plausible interpretation our results.

4. Lines 163-165: it is stated that high lenacapavir levels "presumably" inhibit reverse transcription in THP-1 cells. This can be easily tested by qRT-PCR. Figure 4C (and S2G): it is unclear why the data are expressed as a heat map instead of a bar graph with error bars like all the other data.

This is a good suggestion, and we have now tested how lenacapavir affects reverse transcription in THP-1 cells (new Fig 5A). We find that LEN concentrations above 10 nM begin to inhibit reverse transcription in cells, which agrees with both our in vitro findings (Fig 4A) and with the published IC50 for LEN-mediated inhibition of reverse transcription for GS-CA1 (25 nM; Yant et al., Nat Medicine. 2019. PMID: 31501601).

Regarding our choice of using heat maps to display some of our data, this allows the reader to assess and compare dose-dependent effects in Fig 4C and S2G quickly. According to “best practices” in modern data visualization, heat maps are recommended when showing the relationship between multiple variables in a matrix format, as we have done in these figure panels (please see this collection of data visualization recommendations from Bang Wong: https://mcmanuslab.ucsf.edu/sites/mcmanuslab.ucsf.edu/files/event/file-attachments/data-visualization-nature-methods-selected.pdf). However, we understand the critique from this reviewer that in cases such as in Fig 4C the reader cannot assess biological variability and error in the heat map as presented. Thus, in our revised manuscript we have also provided supplementary bar graphs for these data with error bars, which can be found in new Fig S4C-E.

5. It is surprising that the lack of an effect with or without PQBP1 in the Results was not discussed.

We have now provided a more detailed discussion of our PQBP1 results (and the absence of an effect). Although we did not observe an effect with PQBP1 in vitro or validate that it is required for HIV-1 detection in myeloid cells, we cannot rule out that PQBP1 plays an important role under different circumstances. We hypothesize that interaction with these and other host factors helps to modulate capsid stability and determine the fate of viral cores during entry.

Reviewer #3: 1) Measuring interferon at 48 hours is too late. The investigators should perform a time course using different concentrations of LEN 1-100nM. Investigators should consider that LEN at 10 nM potently inhibit infection. Meaning 10nM or less is the relevant concentration that needs to be considered for interpretation of experiments in cells.

We have now performed a time course to measure activation of IFN signaling in THP-1 cells after infection in the presence and absence of LEN at 10 nM (new Fig 5B). We report that LEN at this concentration potently blocks infection and at the same time, leads to pronounced induction of several ISGs (*IFITM1, IFITM3, MX1, ISG15*, and *IFI27).*

2) It is well-documented by various groups, not cited here, that LEN at 10 nM stabilizes the core; therefore, it should not induce interferon; an stabilized core will not expose the viral genome to sensors. The HIV-1 core is stabilized at 5-10 nM during infection (sufficient amount of drug to potently block HIV-1 infection). Destabilization only occurs at concentrations between 30nM-100nM, which is an excessive

amount of the drug(overkill). Thus, this data is not in agreement with previous findings. The literature should be considered, as two separate papers using the fate of the capsid assay showed stabilization of the core by LEN at 10 nM.

We thank this reviewer for raising this important point. We are beginning to understand that capsid inhibitors in the LEN class stabilize hexamer contacts while destabilizing pentamer contacts. Structural studies have shown that LEN exclusively binds to hexamers and reduces lattice curvature, which is incompatible with closed cone structures. We agree with this reviewer that LEN stabilizes the capsid lattice through hexamer-hexamer bridges and promotes formation of CA tubes in which hexamers have the optimal bite angle. However, LEN does not stabilize closed cones, as it disfavors pentamers, even at the low concentrations of drug. Please see the following references from the labs of Rob Dick, John Briggs, Leo James, Owen Pornillos, Wes Sundquist, Till Boecking, and others, that support these statements and have been incorporated into our manuscript: PMID: 37094124, PMID: 36759579, PMID: 33033190, PMID: 38347802. In our manuscript we have been careful to use language that accurately describes the effects we observe on core integrity, using the terms “disruption” or “integrity” (vs. disassembly) to refer to the loss of core integrity, and we have tried to avoid describing our results in a way that could be misinterpreted as full disassembly of the CA lattice.

Our work broadens understanding of published data and provides the first biochemical evaluation of core integrity. Regarding the fate of the capsid assay, we agree with this reviewer that this assay may be helpful for determining whether capsids or capsid pieces are pelletable, but it does not assess the integrity of a viral core (i.e. whether there are “holes” in the capsid shell that would permit release and/or detection of the viral DNA). We have now cited a publication that we assume this reviewer is referring to that uses the fate of capsid assay (Eschbach et al., mBio. 2024. PMID: 38530034). Contrary to what is suggested in the comment above, our work is consistent with their findings, and further explains how LEN at therapeutic concentrations can expose the viral DNA to innate immune sensors and induce IFN. Eschbach et al., and the Kutluay lab describe how GS-6207 (LEN) and GS-CA1 can block infection while simultaneously allowing for cGAS-mediated sensing of the virus. Their work largely tests high viral titers (MOI of ~5 and higher). In our study, we have rigorously tested different drug concentrations at different virus doses. Our data show that therapeutic concentrations of LEN block infection and either enhance or maintain ISG responses, depending on whether cells are exposed to low or high amounts of virus, respectively (Fig 4; Fig S4; Fig 5).

Thus, our findings extend our understanding of LEN’s mechanism of action, as it progressively disrupts viral core integrity. At low nanomolar concentrations (e.g. 1-10 nM), LEN occupancy is insufficient to disrupt the core completely and reverse transcription proceeds. However, at these concentrations, core integrity is lost, infection is blocked, and the viral DNA becomes accessible to cGAS (Fig S4A-E) (though the molecular details of where in the cell this occurs have yet to be worked out). At higher concentrations of drug (> 30 nM) the integrity of the core is disrupted to the point where reverse transcription is affected (Fig 5A), therefore less viral DNA is available for detection, and IFN responses are diminished. We emphasize that our data are consistent with (and extend the findings of) the Kutluay lab’s recent publication where they used the fate of the capsid assay +/- 10 nM LEN. If there are other specific papers that should be referenced, we would be happy to do so if they are pointed out to us.

3) Investigators should measure core stability during infection in the presence of LEN, and correlate this with induction of ISGs in the particular cells that they are using. These experiments will provide information on whether ISGs are induced upon core destabilization during infection.

As mentioned above, we feel that core "stability" is not the relevant property that pertains to this study, and instead have chosen to focus on developing a method to assess core "integrity" by assaying whether viral DNA is accessible to cGAS. In that regard, we have performed sophisticated measurements of core integrity, and have determined the accessibility of reverse transcription products both in vitro and in cells during infection. By correlating these measurements, our data show that LEN triggers the disruption of core integrity and facilitates cGAS activity in vitro at the same concentrations that ISGs are induced in cells (Fig 4).

4) These experiments were likely performed before the FDA approval of LEN. Therefore, it is crucial to repeat the LEN experiments in cells at all concentrations, conducting a time course to measure ISGs.

We have performed thorough dose response experiments to test LEN and GS-CA1 in THP-1 cells, monocyte-derived dendritic cells, and monocyte-derived macrophages (Fig 4, Fig S4, and Fig 6). At this reviewer's suggestion, we have now also performed a time course experiment to measure ISG expression in THP-1 cells with and without LEN. We find that the kinetics of ISG induction in this study are consistent with published works that include time course experiments of HIV-mediated induction in myeloid cells (Johnson et al., Cell Host & Microbe. 2018. PMID: 29544097; Johnson et al., Cell Reports. 2020. PMID: 31968263; Eschbach et al., mBio. 2024. PMID: 38530034).

5) Figure 4B and C: Measuring the induction of interferon in cells at 48 hours is too late. The investigators should perform a time course using different concentrations of LEN (0-48 hours).

We have done as this reviewer has suggested and performed a time course experiment with LEN from 0 – 48 hours after infection. Since it was stated above that: *"10nM or less is the relevant concentration that needs to be considered for interpretation of experiments in cells,"* we have proceeded to test LEN at 10 nM. We report that ISGs are induced in the temporally expected fashion, with the peak signals observed at 40 h after infection (new Fig 5B), which is consistent with our previous findings (Johnson et al., Cell Host & Microbe 2018. PMID: 29544097). Although the qPCR signal wanes slightly at 48 h, the ISG induction with LEN remains significantly higher than for virus infections performed in the absence of LEN. Since protein expression lags behind RNA expression, measuring ISG expression by flow cytometry at 48 h is actually the ideal time to capture innate immune responses to HIV-1, as we have previously determined (Johnson et al., Cell Reports. 2020. PMID: 31968263).

6) Figure 4B and C: LEN has passed numerous tests, and no reports have demonstrated the induction of ISGs at 10 nM.

It's unclear if this point is framed to be a positive or a negative comment on our study. We note that our manuscript builds on prior work, as we are not the first to demonstrate that capsid inhibitors can potentiate ISGs during HIV-1 infection. Eric Poeschla's lab reported that PF74 could activate a cGAS-STING response during infection (Kumar et al., J Virol. 2018. PMID: 29769349). Greg Towers' lab also demonstrated that PF74 could disrupt capsids and potentiate cGAS sensing of HIV-1 (Sumner et al., Nat Micro. 2020. PMID: 32852081). Both of these studies used the less potent PF74 inhibitor at 10 µM. More recently, Sebla Kutluay's lab published that LEN could increase cGAS-STING responses to HIV-1 (Eschbach et al., mBio. 2024. PMID: 38530034), and showed that ISGs are induced with 10 nM LEN, albeit not to the same degree as in our study, which we speculate to be due to their use of higher virus doses that are already "above threshold" for activating cGAS. Nevertheless, these prior

publications set the precedent that capsid inhibitors can promote innate immune sensing of HIV-1 at therapeutic doses in cells that have the capacity to detect the virus.

7) Figure 4B and C: this figure needs a control using type I IFN in THP-1 cells and measuring the induction of SIGLEC-1.

We understand that in some cases it would make sense to include a positive control to establish that the type I IFN pathway is engaged. However, in this context, including samples with type I IFN would not add any significant information to the experiments in Figure 4B,C. This is because we already see that over 80% of the cells are SIGLEC1-positive with the mean fluorescence intensity of SIGLEC1 expression increasing by more than a log order, so there is not a clear need for a positive control, especially since we have validated that the virus is stimulating the IRF3 pathway (Fig 6). Secondly, the timing required for stimulation and flow cytometry to properly assess responses to type I IFN would need to be very different than for virus infections, so they cannot be fairly compared head-to-head. For these reasons, we do not feel it would be helpful to include samples with type I IFN in Figure 4B and C. If you are curious about the magnitude of this response and would like to see the comparison between virus-driven and recombinant IFN-driven innate immune responses, please see our previous work (Johnson et al., Cell Reports. 2020. PMID: 31968263) which provides a comprehensive resource that catalogues responses to different innate immune agonists (including HIV-1-GFP and recombinant type I IFN).

Additional Comments:

The manuscript is poorly referenced. Many LEN papers are not cited.

We apologize for not providing adequate references. In our revised manuscript we have added 26 additional citations, which now includes a total of 11 papers referencing LEN. If there are specific publications that we should include in addition to these, we'd be happy to do so.

There is a poor description of experimental details in all legends.

We apologize for not describing our experiments well in the figure legends. We have gone through our legends and now provide additional detail where descriptions could have been unclear. Please let us know if there are specific sections that could be further improved.

Line 28: Many references demonstrating RT in the nucleus are missing.

We apologize again for not referencing the literature thoroughly. We have added five additional citations that demonstrate RT in the nucleus, but if these are insufficient, we would appreciate learning more and incorporating additional references where appropriate.

Figure 1D legend: Include ERT sample preparation details and clarify the 16-hour duration.

Experimental details that describe ERT sample preparation are extensive, and so we have provided these in the Methods section. In the legend for Figure 1D, we have clarified that we have performed standard ERT reactions for 16 hours prior to heating samples to the indicated temperatures.

Figure 1E legend: Explain when the change of IP6 concentration occurs. Is it after lysis with Melittin or after the cores have been stabilized in IP6?

The change of IP6 concentration occurs after ERT (otherwise reverse transcription would not proceed, as shown in Fig S2). We now state in the legend that samples from standard ERT reactions were diluted into cell-free sensing assay reactions and incubated with recombinant cGAS under a range of IP6 concentrations.

Revise all legends for accurate descriptions of experiments, including drug concentrations and experiment timelines.

We have taken great care to describe our experiments accurately and have provided drug concentrations and experiment timelines in all cases, in the legends and the Methods section.

Line 100: PF74 at the μM range is quite potent against HIV-1, so correct this statement.

We disagree that PF74 is a "potent" inhibitor, based on our understanding of drug pharmacology. However, we have revised this statement to read: "…this inhibitor has relatively low affinity for the capsid and only blocks viral replication at micromolar concentrations ."

The legend for Figure 4G-H is missing experimental details such as time and drug concentration.

As mentioned above, we have revised our legends to better describe our experiments. The drug concentrations in original Fig 4G-H (new Fig 6D-G) were provided in the figure itself and the timing was listed in the figure legend. If there are additional sections that could be improved, please let us know.

Part III – Minor Issues: Editorial and Data Presentation Modifications
Please use this section for editorial suggestions as well as relatively minor modifications of existing

data that would enhance clarity.

Reviewer #1: The title for this work mentions an already existing and agreed idea. Can this be reworded?

We have revised the title to highlight the advance of our in vitro system: "Cell-free assays reveal that the HIV-1 capsid protects reverse transcripts from cGAS."

Reviewer #2: 1. Lines 67, 315, and 494: pHIVSG3 delta env is not "full-length" if it is lacking the env gene.

We thank this reviewer for drawing our attention to these lines. Our pHIV-SG3 construct contains the *env* gene with several point mutations that disrupt the gene product. Thus, the genome is still "full-length" despite being *env*-deficient, since the measured genome length is the same as wild type virus. We have edited the text to more accurately describe this construct, while maintaining the description of the SG3 genome as "full-length."

2. As timing appears to matter according to Figure 3, how long were the ERT reactions and cGAS sensing assays conducted for Figures 1 and 2B?

For Figures 1 and 2B, ERT reactions were run for 16 h at 37 C. Samples were then transferred to cGAS sensing assays and cGAMP levels were measured after 8 h. The timing of the ERT and cGAS reactions have been described in the Methods Section and are now also listed in the legend for Figure 1 for clarity.

3. Figure S4: it is unclear why elvitegravir was included in the assay.

We apologize for not describing the rationale for the experiment in Fig S4 (now Fig S5 F-G). Elvitegravir was included to inhibit integration in order to limit sensing of virus to the first half of the life cycle, where PQBP1 is reported to play a role. We and others have previously found that a significant proportion of innate immune recognition occurs after integration in myeloid cells (most prominently for monocyte-derived dendritic cells, and this is recapitulated to some degree in THP-1 cells: Manel et al., Nature 2010. PMID: 20829794; Johnson et al., Cell Host & Microbe. 2018. PMID: 29544097). Therefore, we included elvitegravir in a subset of samples with this experiment to prevent post-integration activation of innate immunity and focus on early events where PQBP1 may have a more prominent role. These experiments tell us that under the conditions tested, even when we block integration, PQBP1 is not required for innate immune sensing of HIV-1.

Reviewer #3: N/A
