# Supplementary material for: Cell-free assays reveal that the HIV-1 capsid protects reverse transcripts from cGAS immune sensing
Source: PLoS Pathog. 2025 Jan 28;21(1):e1012206. doi: 10.1371/journal.ppat.1012206 (PMC11793794; doi:10.1371/journal.ppat.1012206)
Supplement: Attachment — Submitted filename: Response to reviewers PLOS Path 20241227.pdf [file ppat.1012206.s009.pdf]

Response to Reviewers

We thank the editor for considering our revised manuscript and thank the reviewers for their comments. Only two minor issues were raised by Reviewer #2. Below, we have responded to these issues with reviewers' comments shown in blue and our responses shown in black.

Dear Dr. Johnson,

Thank you for submitting your manuscript to PLOS Pathogens. After careful consideration, we feel that it has merit but does not fully meet PLOS Pathogens's publication criteria as it currently stands. Therefore, we invite you to submit a revised version of the manuscript that addresses the points raised during the review process.

Reviewer's Responses to Questions:

Part I - Summary

Please use this section to discuss strengths/weaknesses of study, novelty/significance, general execution and scholarship.

Reviewer #1: The authors have satisfactorily addressed the previous review comments. No additional experiments required.

Reviewer #2: My previous comments have been addressed.

Part II – Major Issues: Key Experiments Required for Acceptance

Please use this section to detail the key new experiments or modifications of existing experiments that should be absolutely required to validate study conclusions.

Generally, there should be no more than 3 such required experiments or major modifications for a "Major Revision" recommendation. If more than 3 experiments are necessary to validate the study conclusions, then you are encouraged to recommend "Reject".

Reviewer #1: None

Reviewer #2: None

Part III – Minor Issues: Editorial and Data Presentation Modifications

Please use this section for editorial suggestions as well as relatively minor modifications of existing data that would enhance clarity.

Reviewer #1: None

Reviewer #2: The new title seems incomplete. I would suggest a minor modification: "Cell-free assays reveal that the HIV-1 capsid protects reverse transcripts from cGAS sensing."

This is a good suggestion. We have now entitled our manuscript: *"Cell-free assays reveal that the HIV-1 capsid protects reverse transcripts from cGAS immune sensing."*

Lines 259-260: "CA-binding proteins that play different roles during entry" is not accurate. I would suggest either revising to "nuclear entry" or "early post-entry replication steps".

We thank this reviewer for catching our mistake. This section now reads: "…*cyclophilin A and CPSF6 are well-characterized CA-binding proteins that play different roles during post-entry replication steps and may positively or negatively affect the capsid…"*
